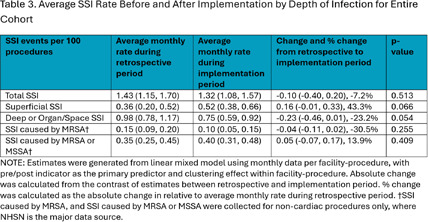# Results From The AHRQ Safety Program for MRSA Prevention: Targeting SSI in High-Risk Surgical Services- Process Measures and Outcomes

**DOI:** 10.1017/ash.2025.238

**Published:** 2025-09-24

**Authors:** Sara Karaba, Melissa Miller, Leyi Lin, Prashila Dullabh, Kathleen Speck, Yue Gao, Jennifer Titus, Sandra Swoboda, Deborah Hobson, Glenn Whitman, Sean Berenholtz, Lisa Maragakis, Roy Ahn

**Affiliations:** 1 Johns Hopkins University School of Medicine; 2 Agency for Healthcare Research and Quality; 3 Johns Hopkins Armstrong Institute for Patient Safety and Quality; 4 NORC at the University of Chicago; 5 JHU School of Medicine; 6 Armstrong Institute for Patient Safety & Quality

## Abstract

**Background:** The Agency for Healthcare Research and Quality Safety Program for MRSA Prevention Surgical Services cohort aimed to reduce surgical site infections (SSIs) and prevent methicillin-resistant Staphylococcus aureus (MRSA) in teams performing surgeries at high risk for infection with and high morbidity due to MRSA (cardiac, knee or hip replacement, and spinal fusion) using evidence-based infection prevention interventions and the Comprehensive Unit-based Safety Program (CUSP) framework. We report process and outcome measures associated with program participation. **Methods:** The Surgical Services Safety Program for MRSA Prevention was implemented from January 2023 to June 2024. The aim was to increase teamwork and collaboration, reinforce safety culture, implement evidence-based infection prevention practices, and decrease SSIs and MRSA. The project team provided 22 live webinars, supporting materials, and other tools to assist surgical teams (Table 1). Teams were also assigned an implementation advisor who provided support through monthly coaching calls.

Teams submitted baseline and endline information on patient safety culture and on infrastructure at the team- and hospital-level, as well as monthly data regarding process measures and SSIs. Teams submitted SSI data from 12 months prior to the start of the program and for 18 months after program implementation. Changes were assessed using pre-post comparisons with Chi-squared test and linear mixed effect models with random intercept. **Results:** 104 surgical teams (18 cardiac, 19 neurosurgical spinal fusion, 16 orthopedic spinal fusion, 51 knee/hip replacement) from 63 hospitals completed the program. Significant improvements in team-based process measures of surgical team infrastructure (Figure 1) and in teams’ reporting that patients received evidence-based practices (Figure 2) were observed across several areas from baseline to endline, including preoperative decolonization, appropriate antibiotic prophylaxis, and intraoperative infection prevention procedures. While SSI rates did not significantly change, the observed 23% decrease in overall deep or organ space SSI rates approached statistical significance (95% CI -0.46, 0.01) (Table 2 and Table 3). **Conclusions:** The AHRQ Safety Program for MRSA Prevention supported implementation of evidence-based infection prevention practices to prevent MRSA and SSIs in high-risk surgeries. Participating teams showed improvements in team-based process measures and observed a reduction in deep or organ space SSI rates.

**Figure:**
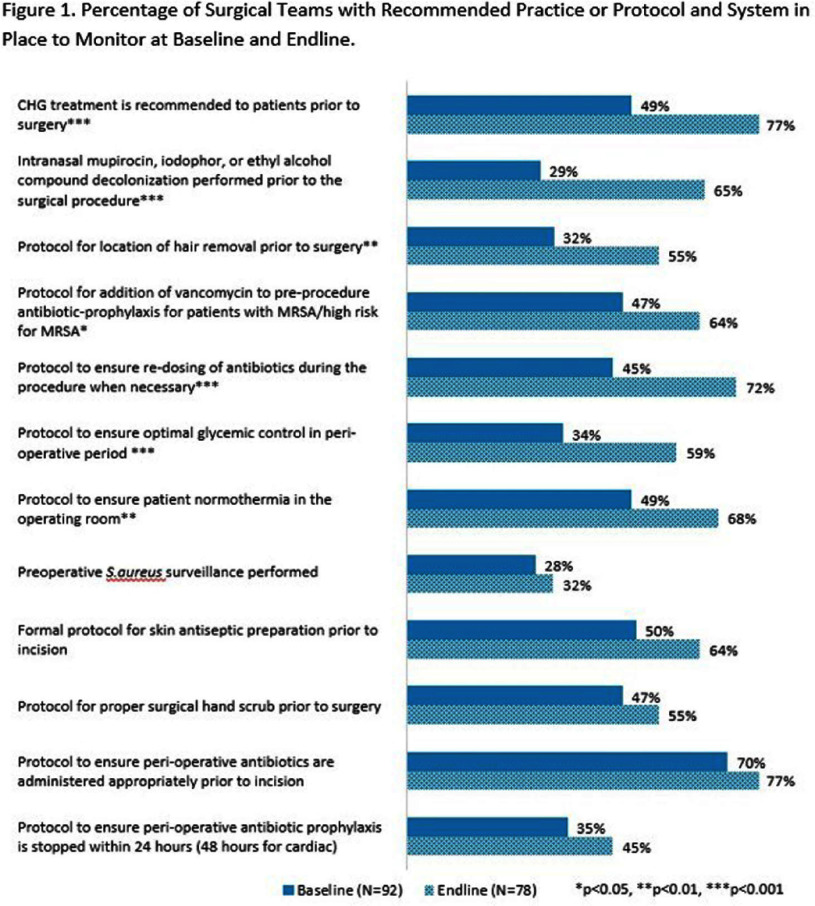

**Figure:**
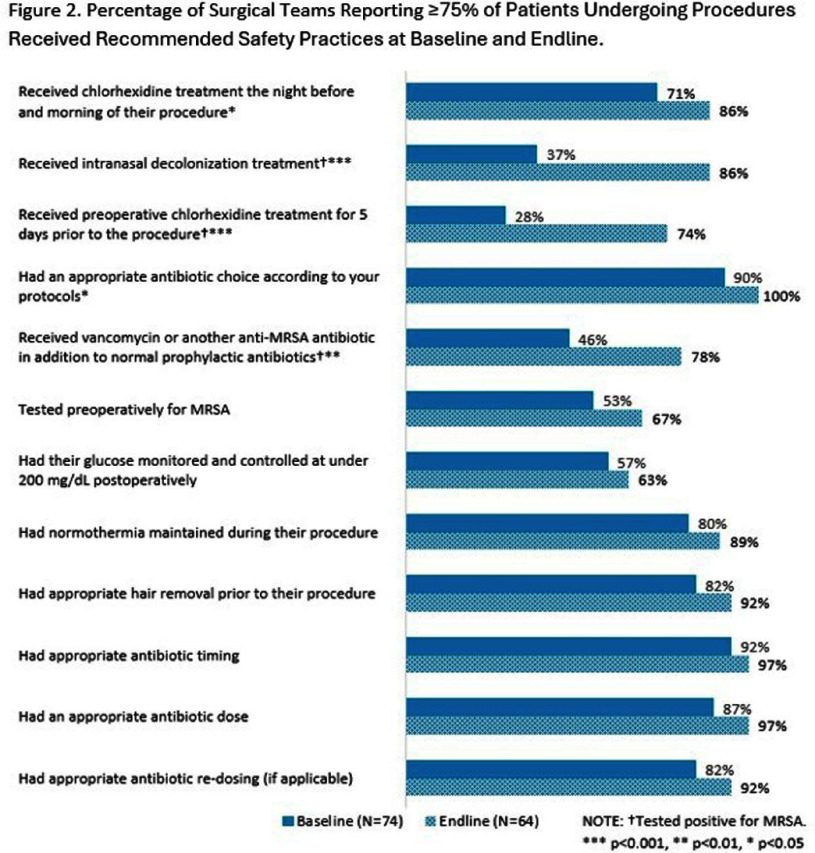

**Figure:**
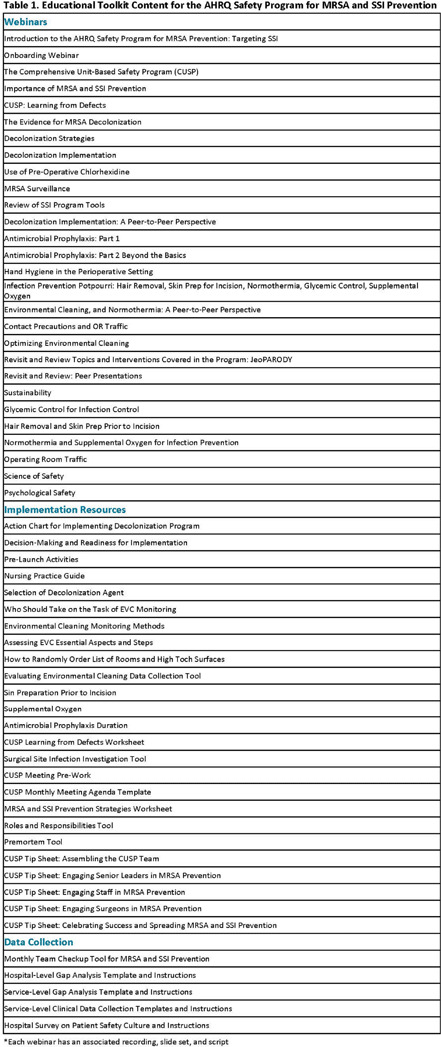

**Figure:**
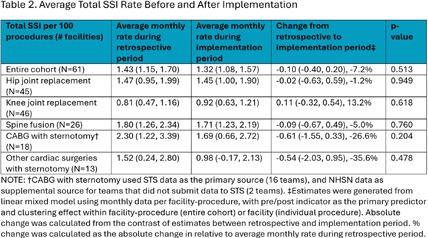

**Figure:**